# Crystallization Products and Structural Characterization of CaO-SiO_2_-Based Mold Fluxes with Varying Al_2_O_3_/SiO_2_ Ratios

**DOI:** 10.3390/ma12020206

**Published:** 2019-01-09

**Authors:** Yuxiang Gao, Mei Leng, Yangfan Chen, Zhichao Chen, Jiangling Li

**Affiliations:** 1 College of Materials Science and Engineering, Chongqing University, Chongqing 400044, China; 20162805@cqu.edu.cn (Y.G.); 201809021083@cqu.edu.cn (M.L.); 20162806@cqu.edu.cn (Y.C.); 20162749@cqu.edu.cn (Z.C.); 2 Chongqing Key Laboratory of Vanadium-Titanium Metallurgical and New Materials, Chongqing University, Chongqing 400044, China

**Keywords:** crystallization products, structure, Al_2_O_3_/SiO_2_ ratio, mold flux

## Abstract

During the casting of high aluminum steel, the dramatic increase in the Al_2_O_3_/SiO_2_ ratio is inevitable, resulting in significant changes of the crystallization behavior, which would result in heat transfer and lubrication problems. Crystallization products and structure characterization of glassy CaO-SiO_2_-based mold fluxes with different Al_2_O_3_/SiO_2_ ratios were experimentally investigated using a differential scanning calorimetry technique and Raman spectroscopy. With increasing Al_2_O_3_/SiO_2_ ratios, the following results were obtained. The crystallization temperature and the crystallization products are changed. With increasing Al_2_O_3_/SiO_2_ ratios from 0.088 to 0.151, the crystallization temperature first increases greatly from 1152 °C to 1354 °C, and then moderately increases. The crystallization ability of the mold flux is strengthened. The species of the precipitated crystalline phase change from two kinds, i.e., Ca_4_Si_2_O_7_F_2_ and Ca_2_SiO_4_, to four kinds, i.e., Ca_4_Si_2_O_7_F_2_, Ca_2_SiO_4_, 2CaO·Al_2_O_3_·SiO_2_ and Ca_12_Al_14_O_32_F_2_, the crystallization ability of Ca_4_Si_2_O_7_F_2_ is gradually attenuated, but other species show the opposite trend. The results of Raman spectroscopy indicate that Al^3+^ mainly acts as a network former by the information of [AlO_4_]-tetrahedral structural units, which can connect with [SiO_4_]-tetrahedral by the formation of new bridge oxygen of Al–O–Si linkage, but there is no formation of Al–O–Al linkage. The linkage of Al–O–Si increases and that of Si–O–Si decreases. The polymerization degree of the network and the average number of bridging oxygens decrease. Further, the relatively strong Si–O–Si linkage gradually decreases and the relatively weak Al–O–Si gradually increases. The change of the crystalline phase was interpreted from the phase diagram and structure.

## 1. Introduction

Due to high strength and good ductility properties, transformation-induced plasticity (TRIP) steel has attracted wide attention for potential applications in automotives [1]. The addition of Al to TRIP steel can contribute to the lightweight of automobiles [2]. However, since the content of [Al] in TRIP steel is much higher than that in ordinary plain carbon steel, it may also cause many problems in practical production [3]. One of the biggest problems is the dramatic change of the chemical composition of the mold flux, especially the Al_2_O_3_/SiO_2_ ratio due to the slag-metal reaction between [Al] in molten steels and SiO_2_ in CaO-SiO_2_-based mold fluxes, as shown in Equation (1) [3]. The increase of Al_2_O_3_/SiO_2_ ratio will significantly modify the physicochemical properties of mold flux such as crystallization, viscosity and melting behavior and would result in problems to control continuous casting [3] because the slab quality is highly related to the physicochemical properties of the flux. 

(1) $${4[\mathrm{Al}]+3\mathrm{SiO}}_{2}{=2\mathrm{Al}}_{2}O_{3}+3[\mathrm{Si}]$$

Crystallization is one of the most important factors determining the heat transfer and lubrication behavior which directly affect the quality of the final steel slabs. The change of crystallization behavior may lead to uneven heat transfer and insufficient lubrication. Cuspidine ($3\mathrm{CaO}\cdot 2{\mathrm{SiO}}_{2}\cdot {\mathrm{CaF}}_{2}$), the main crystalline phase of traditional CaO-SiO_2_-based mold flux, is widely deemed as an optimal crystal to control the heat transfer [4,5,6]. However, during the continuous casting of high aluminum steel, the dramatic increase of the Al_2_O_3_/SiO_2_ ratio is inevitable, resulting in significant changes of the crystallization behavior, which would result in heat transfer and lubrication problems. Therefore, it is necessary to identify how the Al_2_O_3_/SiO_2_ ratio affects the change of the crystallization products. Zhang et al. [7] investigated crystallization with different Al_2_O_3_/SiO_2_ ratios (0.25, 0.75, 1.5, 0.4, mass%) using confocal scanning laser microscopy (CSLM) and found that the crystallization temperature, precipitated phase and crystal morphology changed dramatically. However, their study included only limited Al_2_O_3_/SiO_2_ ratios. 

Crystallization behavior of slags is usually related to the variation of structure [8]. Therefore, the study of the structure will benefit a better analysis of the crystallization of slags. Many techniques have been employed to investigate the slag structure, such as Raman spectroscopy [9], nuclear magnetic resonance (NMR) [9], and infrared characteristic absorption spectrum (IR) [10], etc. Previous studies mainly focused on the structure of the mold flux, whereas very few studies were related to its correlation with the crystallization variation caused by the change of the Al_2_O_3_/SiO_2_ ratio. Cui et al. [10] studied the effect of the SiO_2_/Al_2_O_3_ ratio on blast furnace slag by an infrared characteristic absorption spectrum and showed that the silicates mainly exist in [SiO_4_]-tetrahedra, while the aluminum atoms are in different coordination states, and the bonding strengths rise with increasing SiO_2_/Al_2_O_3_ ratio. Liao et al. [11] studied the effect of the Al_2_O_3_/SiO_2_ ratio (varied from 0.11 to 0.8) on the structure of CaO-SiO_2_-MgO-Al_2_O_3_ slag and found that the degree of polymerization for [SiO_4_]-tetrahedra decreases with increasing Al_2_O_3_/SiO_2_ ratio, based on Fourier Transformation-Infrared Spectroscopy (FT-IR) and Raman spectroscopy. In order to better understand the change of crystallization products, it is necessary to study the dependence of structure and crystallization on various Al_2_O_3_/SiO_2_ ratios of mold fluxes. 

In the current work, the change of crystallization products with a variation of the Al_2_O_3_/SiO_2_ ratio was investigated using a differential scanning calorimeter (DSC) combined with X-ray diffraction (XRD) and scanning electronic microscopy (SEM). In addition, the Raman technique was applied to study the structure of the glassy mold fluxes. The correlation between the crystallization and structure of the CaO-SiO_2_-based mold fluxes is discussed.

## 2. Materials and Methods

### 2.1. Sample Preparation

Powder mold fluxes were designed with different Al_2_O_3_/SiO_2_ ratios based on the chemical composition change during the casting process of high Al steel. Five samples were used, and their chemical compositions in weight% are listed in Table 1. Reagent grade CaCO_3_, CaF_2_, Na_2_CO_3_, SiO_2_ and Al_2_O_3_ were used to prepare the synthetic mold fluxes. To obtain high purity of CaO, the CaCO_3_ was calcined at 1373 K for 7 h in a muffle furnace to obtain CaO identified by the fore and aft weightlessness. 

**Figure 1:**
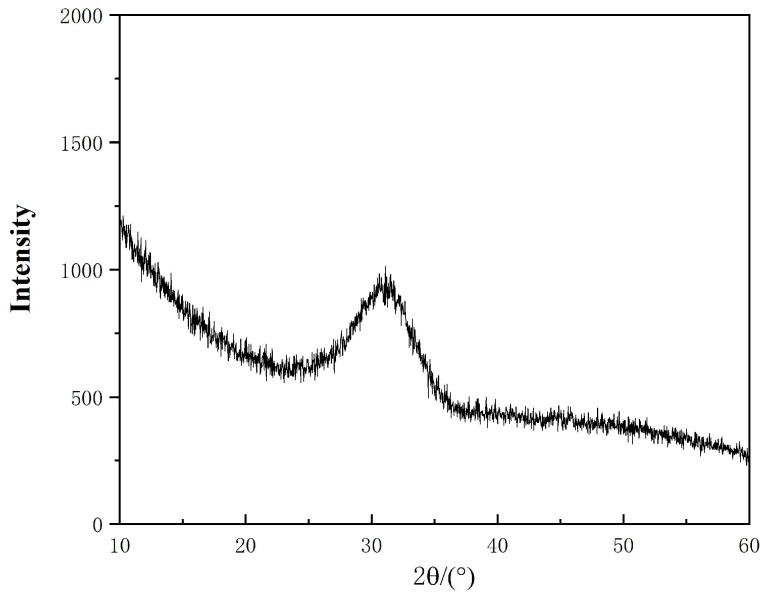
X-ray diffraction patterns of glassy mold flux (Sample No. 1).

The glassy samples were prepared by regular melting and quenching methods. The samples were first put into a platinum crucible and melted in a high temperature tube furnace with a heating element in molybdenum silicide at 1723 K (1450 °C), and then, the molten mold fluxes were quenched in water to form glasses as identified by XRD in Figure 1. 

### 2.2. Differential Scanning Calorimetry Analysis

The glassy mold fluxes were ground into powders with a size less than 250 μm in diameter and were put into platinum crucibles for measurement by differential scanning calorimetry (DSC). The DSC measurement was performed in an argon atmosphere in the temperature range of 673–1723 K (or 400–1450 °C) using a Netzsch DSC404 F3 calorimeter (Netzsch Corporation, Selb, Germany). The cooling rate of all measurements was 5 K/min. α-Al_2_O_3_ was used as a reference material in the present experiments. 

### 2.3. X-ray Diffraction and Scanning Electron Microscope Analysis

The phases and crystal morphologies of the crystallized mold fluxes were subjected to X-Ray Diffraction (XRD) analysis and a Scanning Electron Microscope equipped with an energy dispersive X-ray spectroscopy (SEM–EDS) microanalyzer. X-ray diffraction experiments were conducted on a 18KW X-ray diffractometer (RIGAKU TTR III, Rigaku Corporation, Tokyo, Japan). The SEM-EDS examinations were carried out using TESCAN VEGA 3 LMH (TESCAN Corporation, Brno, Czech Republic). 

### 2.4. Raman Spectroscopy Analysis

In order to understand the effect of the Al_2_O_3_/SiO_2_ ratio on the structure of CaO-SiO_2_-based mold flux, the glassy samples were analyzed using nonpolarized Raman spectroscopy measurements. All samples were recorded in the frequency range of 300–3000 cm^−1^ at room temperature. The excitation wavelength of 532 nm and a semiconductor laser with power of 1 mW in a micro-Raman spectrometer made in France (LabRAM HR Evolution, HORIBA Jobin Yvon) were used. The frequency range was mainly between 400 and 1500 cm^−1^ for all samples. The Raman spectra were fitted using peak-fit software by assuming Gaussian functions to obtain more specific structure information. The areas of the deconvoluted peaks were calculated by the software to evaluate the change of the network polymerization of the glassy mold flux.

## 3. Results and Discussion

### 3.1. Crystallization Analysis of CaO-SiO_2_-Based Mold Fluxes

**Figure 2:**
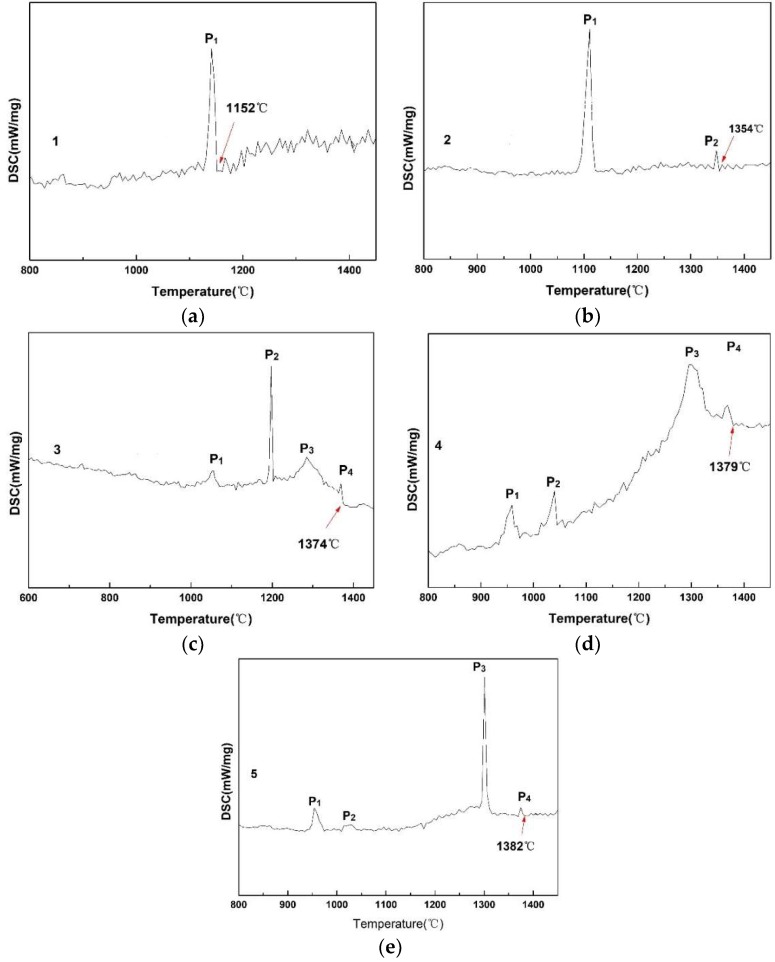
DSC results of the non-isothermal crystallization process of the CaO-SiO_2_ mold fluxes with increasing Al_2_O_3_/SiO_2_ ratio. (**a**) Al_2_O_3_/SiO_2_ = 0.088; (**b**) Al_2_O_3_/SiO_2_ = 0.151; (**c**) Al_2_O_3_/SiO_2_ = 0.261; (**d**) Al_2_O_3_/SiO_2_ = 0.367; (**e**) Al_2_O_3_/SiO_2_ = 0.562.

**Figure 3:**
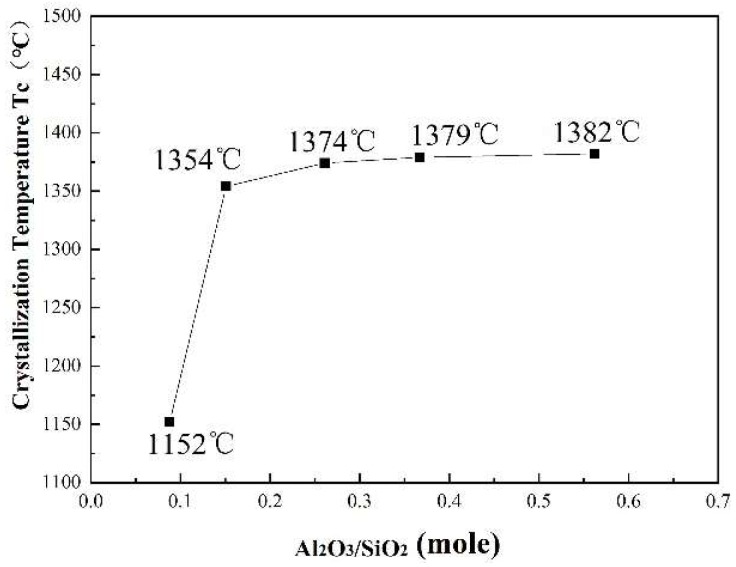
Change of the crystallization temperature of the mold fluxes with increasing Al_2_O_3_/SiO_2_ ratio.

Figure 2 shows the results of DSC measurements of the CaO-SiO_2_-based mold fluxes with varied Al_2_O_3_/SiO_2_ ratios. It is observed that with increasing Al_2_O_3_/SiO_2_ ratio, the number of exothermic peaks on the DSC curves increases. Only one obvious exothermic peak for Sample 1 with Al_2_O_3_/SiO_2_ = 0.147 is detected, and the number of exothermic peaks gradually increases from 1 to 4 with the Al_2_O_3_/SiO_2_ ratio increasing from 0.088 to 0.562, which indicates that crystallization events gradually increase from one to four. Alternatively, the exothermic peaks move towards high temperatures, which suggests that the crystallization temperatures are increased and the crystallization ability of the CaO-SiO_2_-based mold flux is enhanced [10]. The specific change in the crystallization temperature is shown in Figure 3. The crystallization temperature first greatly increases from 1152 °C to 1354 °C with the Al_2_O_3_/SiO_2_ mole ratio increasing from 0.088 to 0.151, and then the temperature increases less with the further increase in the Al_2_O_3_/SiO_2_ ratio. This finding indicates that with increasing Al_2_O_3_/SiO_2_ ratio, the crystallization temperature tends to raise, especially at low Al_2_O_3_/SiO_2_ ratios. Therefore, the increase of Al_2_O_3_/SiO_2_ ratio can improve the crystallization ability of the CaO-SiO_2_-based mold flux. Similarly, Zhang et al. [7] observed the crystallization behavior in mold slags and found that with increasing Al_2_O_3_/SiO_2_ ratio, the crystallization temperature and crystallization ability increased. 

**Figure 4:**
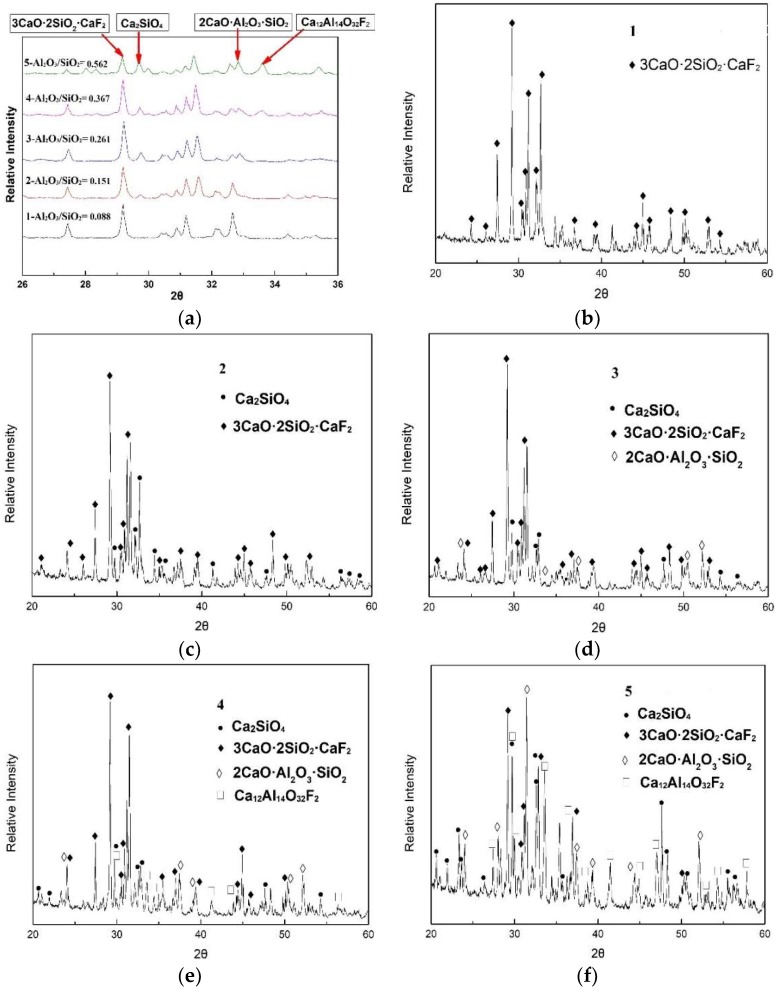
Phase identified by XRD for the CaO-SiO_2_-based mold flux. (**a**) The overall comparison diagram; (**b**) Al_2_O_3_/SiO_2_ = 0.088; (**c**) Al_2_O_3_/SiO_2_ = 0.151; (**d**) Al_2_O_3_/SiO_2_ = 0.261; (**e**) Al_2_O_3_/SiO_2_ = 0.367; (**f**) Al_2_O_3_/SiO_2_ = 0.562.

In order to identify the various specific crystallization products, the crystallized mold fluxes were analyzed using XRD and SEM-EDS techniques. The heat treatment experiments were performed to determine the phase precipitation using the same cooling rate (5 K/min) because of the small samples after the DSC measurements for XRD and SEM analysis. XRD analysis of the precipitated crystalline products is shown in Figure 4a–f. From Figure 4a, three obvious changes in the characteristic peaks of XRD can be revealed as labeled in the picture. First, the cuspidine (Ca_4_Si_2_O_7_F_2_) crystal precipitated in all the samples and the relative amount of cuspidine decrease with increasing Al_2_O_3_/SiO_2_ ratio. Only cuspidine (Ca_4_Si_2_O_7_F_2_) crystal precipitates when Al_2_O_3_/SiO_2_ = 0.147 (CaO/SiO_2_ = 1). Previous reports obtained similar results for the precipitated crystalline phase of the traditional mold flux for CaO/SiO_2_ = 1 [4,12,13]. In addition, the new crystalline phases Ca_2_SiO_4_, 2CaO·Al_2_O_3_·SiO_2_ and Ca_12_Al_14_O_32_F_2_ gradually precipitate with increasing Al_2_O_3_/SiO_2_ ratio from 0.258 to 0.950. The specific XRD results for each sample are shown in Figure 4b–f. When Al_2_O_3_ gradually increases and SiO_2_ gradually decreases, Ca_2_SiO_4_ precipitates due to the increase in the CaO/SiO_2_ ratio. Watanabe. T et al. [6] investigated the phase equilibria of solid and liquid coexisting 50.8 mass% CaO-38.6 mass% SiO_2_-10.6 mass% CaF_2_ and also found that the cuspidine and Ca_2_SiO_4_ co-precipitate. When the Al_2_O_3_/SiO_2_ ratio increases to 0.261, cuspidine, Ca_2_SiO_4_ and 2CaO·Al_2_O_3_·SiO_2_ co-precipitate. Our previous study on phase relations in CaO-SiO_2_-Al_2_O_3_-15% CaF_2_ slags found that 2CaO·Al_2_O_3_·SiO_2_ is produced with an increasing content of Al_2_O_3_ in the mold flux [13]. When the Al_2_O_3_/SiO_2_ ratio further increases, a new crystalline phase of Ca_12_Al_14_O_32_F_2_ precipitates. The precipitated cusipidine gradually decreases, indicating that the content of fluorine and the amount of Al_2_O_3_ increase in the molten fluxes is caused by the increase in the Al_2_O_3_/SiO_2_ ratio. The increase of fluorine and Al_2_O_3_ induce the formation of Ca_12_Al_14_O_32_F_2_. From the relative intensity of the XRD characteristic peak, it can be found that the crystallization ability of cuspidine decreases with increasing Al_2_O_3_/SiO_2_ ratio, and the other species show the opposite trend.

**Figure 5:**
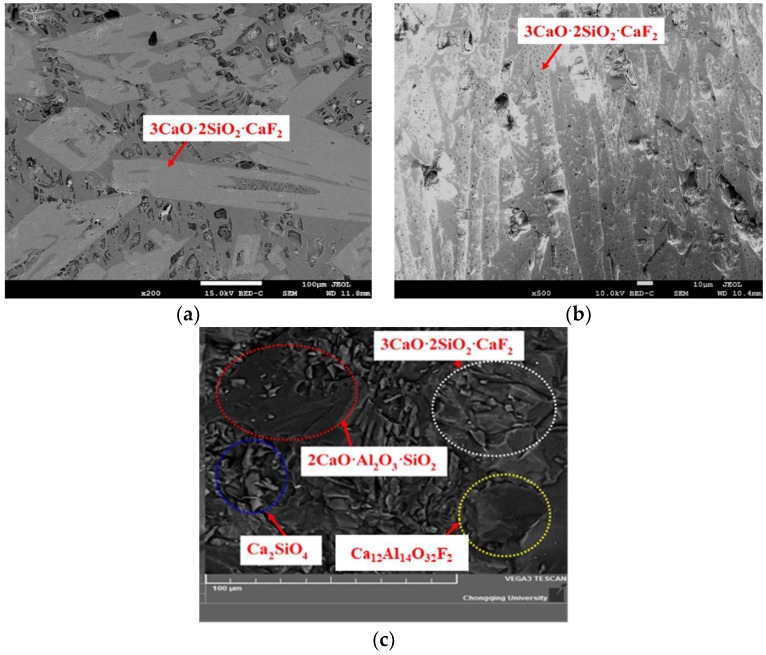
Phase identified by SEM-EDS for the CaO-SiO_2_-based mold fluxes. (**a**) No.1; (**b**) No.2; (**c**) No.5.

Block samples were taken for SEM-EDS analysis to determine the phase and crystal morphology. The mold fluxes appeared severely pulverized with the increase in the Al_2_O_3_ content. When Al_2_O_3_/SiO_2_ ratio increases to 0.261, no block can be formed. Therefore, Samples 1 and 2 were observed by SEM-EDS to identify the phase and crystal morphology. Powder sample No. 5 was also analyzed by SEM-EDS to identify the phase. The results are shown in Figure 5a–c; it can be observed that cuspidine (Ca_4_Si_2_O_7_F_2_) has a lath-like or faceted morphology in Samples 1 and 2, and in Sample 2, Ca_2_SiO_4_ could not be found, perhaps due to the small size and low amount. Guo et al. [14] reported that cuspidine presents the same morphology as in traditional CaO-SiO_2_ mold flux. Four types of crystalline phases are identified in the SEM of Sample 5, which is consistent with the XRD results.

It can be concluded from the above results that the species of the precipitated phase change in the following sequence with increasing Al_2_O_3_/SiO_2_ ratio: from Ca_4_Si_2_O_7_F_2_ to Ca_2_SiO_4_ and Ca_4_Si_2_O_7_F_2_, and then to Ca_2_SiO_4_, 2CaO·Al_2_O_3_·SiO_2_ and Ca_4_Si_2_O_7_F_2_, and finally to Ca_2_SiO_4_, 2CaO·Al_2_O_3_·SiO_2_, Ca_4_Si_2_O_7_F_2_ andCa_12_Al_14_O_32_F_2_. Cuspidine, as the main crystallization product of the traditional CaO-SiO_2_-based mold flux, is strongly deemed as the optimal crystal to control the heat transfer and lubrication during casting [4]. However, many new types of Al_2_O_3_-containing crystals precipitate with increasing Al_2_O_3_/SiO_2_ ratio, resulting from the slag-steel reaction during high aluminum steel casting. It was reported that many horizontal and vertical depressions containing open cracks appeared on the surface of the slabs when using CaO-SiO_2_-based mold fluxes during high aluminum steel casting [15]. Our current work focuses on the change of crystallization products caused by the changes in the chemical composition caused by the slag-steel reaction, which contributes to the understanding of high aluminum steel casting problems.

### 3.2. Structural Analysis of CaO-SiO_2_-Based Mold Fluxes

**Figure 6:**
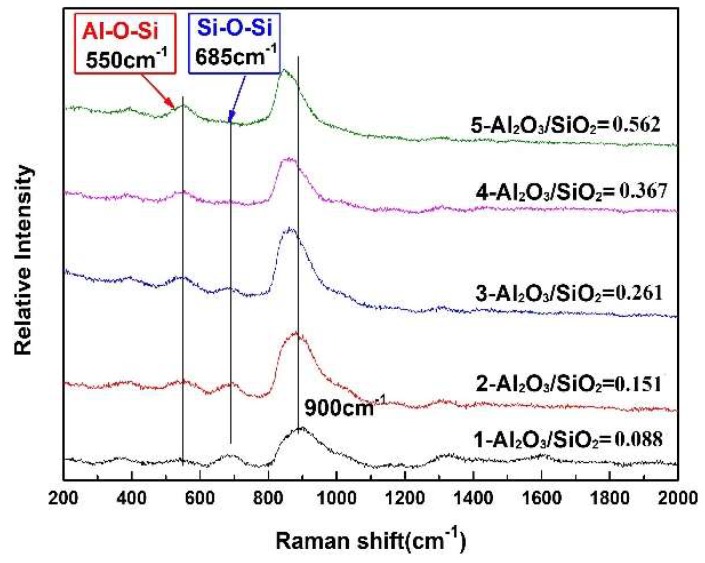
Raman spectra for glassy samples with different Al_2_O_3_/SiO_2_ ratios.

The Raman spectra of the glassy samples with varying Al_2_O_3_/SiO_2_ ratio are presented in Figure 6. It can be seen that with increasing Al_2_O_3_/SiO_2_ ratio, the intensity of the Raman bands first gradually increases at lower frequency between 450–600 cm^−1^, and then decreases between 600–800 cm^−1^. According to previous reports [16,17,18], a Raman frequency range between 450–600 cm^−1^ corresponds to mixed bending and stretching vibration of the Al–O–Si bridge oxygen linkage, and a Raman frequency range between 600–800 cm^−1^ is a signature of mixed bending and stretching vibration of the Si–O–Si bridge oxygen linkage. Therefore, it can be concluded that the bridge oxygen linkage of Al–O–Si gradually increases and the bridge oxygen linkage of Si–O–Si decreases with increasing Al_2_O_3_/SiO_2_ ratio. In the present system, Al_2_O_3_ predominantly acts as a network in the formation of [AlO_4_]-tetrahedral structural units due to the presence of significant basic oxides such as CaO and Na_2_O [17]. In aluminosilicate glasses and melts, [AlO_4_] can be stabilized by the proximity of M^2+/+^ ions. The M^2+^ or M^+^ ions in excess will destroy the aluminosilicate glasses network structure so as to increase the formation of non-bridging oxygens (NBOs) [19]. In the present mold flux glasses, the value of the (M^2+^ + M^+^)/Al_2_O_3_ ratio is much higher than 1, so that Al^3+^ mainly forms [AlO_4_] to participate in the formation of the silicate network. Based on Loewenstein rules [20], one aspect of the short-range order of framework cations can be expressed as Al avoidance, which postulates that the Al–O–Si linkage is more favorable than the combinations of Si–O–Si and Al–O–Al. That is to say, the Al–O–Si linkage forms primarily in aluminosilicate glasses [20]. This is consistent with the present result: the bridge oxygen linkage of Al–O–Si increases and bridge oxygen linkage of Si–O–Si decreases with increasing Al_2_O_3_/SiO_2_ ratio. Since the mole content of SiO_2_ is much higher than that of Al_2_O_3_ in all samples, [AlO_4_] tends to form the Al–O–Si linkage. Therefore, it can be concluded that [AlO_4_] and [SiO_4_] as network former units form a network structure and two kinds of bridge oxygen linkages (Al–O–Si and Si–O–Si) appear in the investigated mold fluxes. Besides, the amount of Al–O–Si linkage increases and that of Si–O–Si linkage decreases with increasing Al_2_O_3_/SiO_2_ ratio.

**Figure 7:**
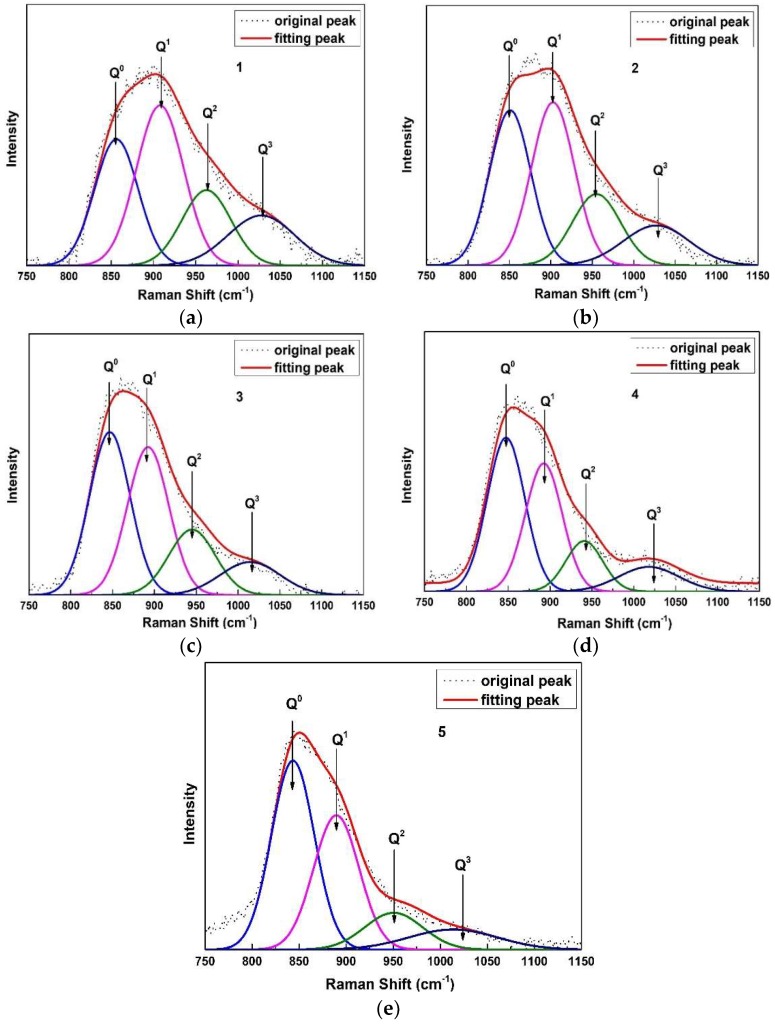
Deconvolved results of Raman spectral for samples with different Al_2_O_3_/SiO_2_ ratios. (**a**) Al_2_O_3_/SiO_2_ = 0.088; (**b**) Al_2_O_3_/SiO_2_ = 0.151; (**c**) Al_2_O_3_/SiO_2_ = 0.261; (**d**) Al_2_O_3_/SiO_2_ = 0.367; (**e**) Al_2_O_3_/SiO_2_ = 0.562.

It can be observed at a higher frequency range (800–1100 cm^−1^) that the Raman shift associated with the vibrations of several depolymerized silicate and aluminosilicate units moves toward lower frequency. For the higher frequency band, the Raman spectra was deconvoluted using the Gaussian-Fitting method similar to that used by Mysen et al. [21]. The deconvoluted results are shown in Figure 7a–e. It can be observed that there are four characteristic bands near 850 cm^−1^, 900 cm^−1^, 950 cm^−1^ and 1030 cm^−1^, which correspond to Q^0^, Q^1^, Q^2^ and Q^3^ with BO/Al = 0, 1, 2, 3, respectively, in the silicate glasses based on the previous references [21,22,23,24]. As shown in Figure 7a–e, it could be found that the full width at half maximum gradually decreases from 136 to 130, 112, 101 and 93 cm^−1^ with increasing Al_2_O_3_/SiO_2_ ratio, inferring that the glass formation ability was impaired. This can further explain the enhancement of crystallization ability of the CaO-SiO_2_-based mold flux with increasing Al_2_O_3_/SiO_2_ ratio.

**Figure 8:**
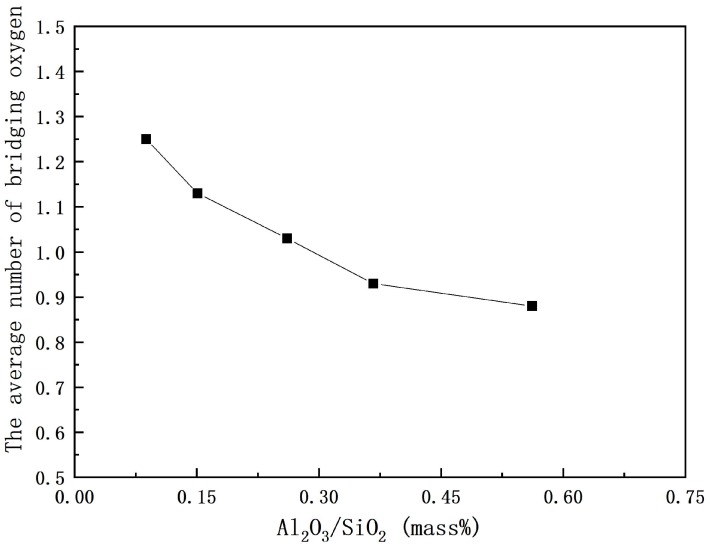
Effect of the Al_2_O_3_/SiO_2_ ratio on the average number of bridging oxygens of glassy mold fluxes.

The proportion of each structure unit can be evaluated by the corresponding integrated areas [4]. Detailed quantitative deconvolution of Raman bands is listed in Table 2. Q^0^ increases with increasing Al_2_O_3_/SiO_2_ ratio, but Q^1^, Q^2^ and Q^3^ slightly decrease. This means that the higher number of bridge oxygen structure units decreases during the process, indicating that the polymerization degree of the network is reduced. In the present work, the average number of bridging oxygens of each sample (N^i^, which can be estimated by the area ratio of each structural units (Q^n^, n = 0, 1, 2, 3) multiplied by the number of its bridging oxygen, i is the sample number) is used to explain the change of the silicate structural network shown in Figure 8 [4]. It can be observed that the average number of bridging oxygens decreases from 1.25 to 0.88, which may be caused by the decrease of the total molar content of Al_2_O_3_ and SiO_2_.

This change of the structure inevitably changes the crystallization behavior. With increasing Al_2_O_3_/SiO_2_ ratio, the polymerization degree of the network and the average number of bridging oxygen decrease. The bond strength of Al–O (330–422 kJ/g atom) [25] is weaker than the Si–O bond (443 kJ/g atom) [25], which causes relatively weaker connections of the network of mold flux with the increase of the Al_2_O_3_/SiO_2_ ratio. These two reasons together would generate a lower energy barrier for ions transferring from bulk glass to glass-crystal interface during crystallization, leading to increasing crystallization ability with increasing Al_2_O_3_/SiO_2_ ratio. From another perspective, the increased amount of Al–O–Si linkage in molten slag would increase the similarity between the molten slag and crystals containing both Si and Al, which would induce the precipitation of the 2CaO·Al_2_O_3_·SiO_2_ crystal containing both Si and Al from CaO-SiO_2_-CaF_2_ system. Q^1^ is the dominant unit in Sample 1, which is the traditional mold flux in which increasing an Al_2_O_3_/SiO_2_ ratio leads to the decreased amount of Q^1^. According to a previous study by Saburi et al. [26], the primary structure unit of cuspidine is Q^1^. With increasing Al_2_O_3_/SiO_2_ ratio, the gradual decrease in the amount of Q^1^ in CaO-SiO_2_-based mold flux would decrease the similarity between the molten slag and cuspidine, so as to reduce the nucleation and growth of cuspidine. 

## 4. Conclusions

During the casting of high aluminum steel, the dramatic increase of the Al_2_O_3_/SiO_2_ ratio is inevitable, resulting in significant changes of the crystallization behavior, which would result in heat transfer and lubrication problems. Crystallization products and structural characterization of glassy CaO-SiO_2_-based mold fluxes with different Al_2_O_3_/SiO_2_ ratios were investigated by the DSC technique and Raman spectroscopy. With the increase of the Al_2_O_3_/SiO_2_ ratio in mold fluxes, the conclusions can be summarized as follows.

(1) The crystallization temperature and the crystallization products have been changed. The crystallization greatly increases from 1152 °C to 1354 °C with the Al_2_O_3_/SiO_2_ ratio changing from 0.147 to 0.258, and then it increases slowly. The crystalline phases are increased from two kinds (Ca_4_Si_2_O_7_F_2_ and Ca_2_SiO_4_) to four kinds (Ca_4_Si_2_O_7_F_2_, Ca_2_SiO_4_, 2CaO·Al_2_O_3_·SiO_2_ and Ca_12_Al_14_O_32_F_2_). The crystallization ability of cuspidine decreases, but the other species show the opposite trend.
(2) Two types of bridge oxygen linkages, i.e., Al–O–Si and Si–O–Si, are formed in CaO-SiO_2_-based mold fluxes. The polymerization degree of the network and the average number of bridging oxygens decrease. The relatively strong Si–O–Si linkage gradually decreases and the relatively weak Al–O–Si bond gradually increases, which cause the weaker link of the molten fluxes.
(3) The gradual increase of the weaker Al–O and the decrease in the amount of the stronger Si–O bond, which causes the relatively weaker connections of the network of the mold flux, give rise to the lower energy barrier for ions transferring from bulk glass to the glass-crystal interface during crystallization. Consequently, the crystallization ability increases.
(4) The increase in the Al–O–Si linkage in molten slag would increase the similarity between the molten slag and crystals containing both Si and Al, which would induce the precipitation of 2CaO·Al_2_O_3_·SiO_2_ crystal containing both Si and Al from the CaO-SiO_2_-CaF_2_ system. The gradual decrease in the amount of Q^1^ in the CaO-SiO_2_-CaF_2_-based mold flux would decrease the similarity between the molten slag and cuspidine and reduce the nucleation and growth of cuspidine.

## Figures and Tables

**Table 1:** The chemical compositions of CaO-SiO_2_-based mold flux with the variation of the Al_2_O_3_/SiO_2_ ratio.

Sample No.	Composition (mass%/mole%)					
	CaO	SiO_2_	Al_2_O_3_	CaF_2_	Na_2_O	Al_2_O_3_/SiO_2_
1	38.0/41.9	34.0/35.1	5.0/3.1	15.0/11.9	8.0/8.0	0.147/0.088
2	38.0/42.5	31.0/32.4	8.0/4.9	15.0/12.1	8.0/8.1	0.258/0.151
3	38.0/43.3	27.0/28.7	12.0/7.5	15.0/12.3	8.0/8.2	0.444/0.261
4	38.0/43.9	24.0/25.9	15.0/9.5	15.0/12.4	8.0/8.3	0.625/0.367
5	38.0/44.7	20.0/21.9	19.0/12.3	15.0/12.6	8.0/8.5	0.950/0.562

**Table 2:** Deconvolved results of Raman spectra for CaO-SiO_2_-based glasses.

Sample No.	Q^0^	Q^1^	Q^2^	Q^3^	N^i^
1	27.10	37.56	18.46	16.88	1.25
2	32.63	35.94	17.70	13.73	1.13
3	37.03	34.43	17.26	11.28	1.03
4	41.62	34.16	13.46	10.77	0.93
5	44.46	33.67	11.86	10.01	0.88
